# Supplementary material for: Autophagy induction mitigates FUS aggregate formation and early synaptic dysfunction at the NMJ in a FUS-ALS model
Source: Cell Death Discov. 2026 Aug 14;12:451. doi: 10.1038/s41420-026-03286-6 (PMC13752260; doi:10.1038/s41420-026-03286-6)
Supplement: Supplementary file 1 [file 41420_2026_3286_MOESM3_ESM.docx]

**Supplementary Fig. 1: p62 accumulation at the NMJ in the FUS^R521C^ model is rescued with rapamycin treatment**

(A-C’) P62 accumulation at the NMJ with the expression FUS^WT^ (A-A’), FUS^R521C^ (B-B’), and FUS^R521C^ on rapamycin-supplemented food (C-C’), p62 (magenta) and HRP (green). (D) p62 intensity for all three genotypes. **p<0.01 and ****p<0.0001 using one-way ANOVA with Tukey’s Post hoc test for multiple comparisons.  Scale Bar in C’’ is 10μm.

**Supplementary Fig. 2: Blocking GluRs abolishes EJPs in control flies and recovered after washout**

(A-C’) EJPs in control flies and the 1^st^ 9 EJPs before (A-A’), during DNQX application (B-B’) , and 10 mins after washout(C-C’).
